# The DNA damage response promotes polyomavirus JC infection by nucleus to cytoplasm NF- kappaB activation

**DOI:** 10.1186/s12985-017-0707-7

**Published:** 2017-02-15

**Authors:** Martyn K. White, Anna Bellizzi, Gabriele Ibba, Valeria Pietropaolo, Anna T. Palamara, Hassen S. Wollebo

**Affiliations:** 1 Center for Neurovirology, Department of Neuroscience, Lewis Katz School of Medicine at Temple University, 3500 N. Broad Street, Philadelphia, PA 19140 USA; 2 Department of Public Health and Infectious Diseases, Institute Pasteur Italia, Cenci-Bolognetti Foundation, Sapienza University of Rome, 5 P.le Aldo Moro, 00185 Rome, Italy; 3 Department of Public Health and Infectious Diseases, Sapienza University, 5 P.le Aldo Moro, 00185 Rome, Italy; 4 San Raffaele Pisana IRCCS, Telematic University, Rome, Italy

**Keywords:** Progressive multifocal leukoencephalopathy, Polyomavirus JC, DNA damage response, Nuclear factor kappa-B

## Abstract

**Background:**

Infection of glial cells by human neurotropic polyomavirus JC (JCV), the causative agent of the CNS demyelinating disease progressive multifocal leukoencephalopathy (PML), rapidly inflicts damage to cellular DNA. This activates DNA damage response (DDR) signaling including induction of expression of DNA repair factor Rad51. We previously reported that Rad51 co-operates with the transcription factor NF-κB p65 to activate JCV early transcription. Thus Rad51 induction by JCV infection may provide positive feedback for viral activation early in JCV infection. DDR is also known to stimulate NF-κB activity, a phenomenon known as nucleus to cytoplasm or “inside-out” NF-κB signaling, which is initiated by Ataxia telangiectasia mutated (ATM) protein, a serine/threonine kinase recruited and activated by DNA double-strand breaks. Downstream of ATM, there occurs a series of post-translational modifications of NF-κB essential modulator (NEMO), the γ regulatory subunit of inhibitor of NF-κB (IκB) kinase (IKK), resulting in NF-κB activation.

**Methods:**

We analyzed the effects of downstream pathways in the DDR by phosphospecific Western blots and analysis of the subcellular distribution of NEMO by cell fractionation and immunocytochemistry. The role of DDR in JCV infection was analyzed using a small molecule inhibitor of ATM (KU-55933). NEMO sumoylation was investigated by Western and association of ATM and NEMO by immunoprecipitation/Western blots.

**Results:**

We show that JCV infection caused phosphorylation and activation of ATM while KU-55933 inhibited JCV replication. JCV infection caused a redistribution of NEMO from cytoplasm to nucleus. Co-expression of JCV large T-antigen and FLAG-tagged NEMO showed the occurrence of sumoylation of NEMO, while co-expression of ATM and FLAG-NEMO demonstrated physical association between ATM and NEMO.

**Conclusions:**

We propose a model where JCV infection induces both overexpression of Rad51 protein and activation of the nucleus to cytoplasm NF-κB signaling pathway, which then act together to enhance JCV gene expression.

## Background

The human neurotropic polyomavirus JC (JCV) infects the oligodendrocytes and astrocytes of the central nervous system (CNS) causing expanding regions of demyelination within the white matter that are involved in the pathogenesis of the fatal demyelinating disease known as progressive multifocal leukoencephalopathy (PML) [1]. Primary infection by JCV is very common and most people are seropositive for JCV beginning at an early age [2]. In contrast, the occurrence of PML is rare and is the result of reactivation of persistent virus usually associated with severe immune dysregulation, which leads to productive infection of glial cells and onset of PML [3]. Several types of immune dyfunction can predispose to the onset of PML and among the most common are HIV-1/AIDS [4] and treatment with therapeutic immunomodulatory drugs used for autoimmune diseases, e.g., natalizumab in multiple sclerosis patients [5, 6]. Our understanding of the molecular events of the JCV life cycle including the reactivation of persistent virus and the pathogenesis of PML is continuing to evolve [2, 3, 7].

JCV is a small DNA tumor virus belonging to the polyomavirus family that has a ~5.1 Kbp, circular, closed, supercoiled, double-stranded DNA genome. Both JCV and Polyomavirus BK (BKV), the causative agent of BKV-associated nephropathy, were discovered in 1971 and for decades they were the only known human polyomaviruses [1, 8]. About 6 years ago, a series of novel polyomaviruses began to be discovered and this has brought the number of human polyomaviruses that are now known to at least ten [9]. The JCV genome is comprised of two coding regions, early and late, separated by a noncoding control region (NCCR), which contains promoter/enhancer elements and the viral origin of DNA replication, Ori [10]. The early region encodes large T-antigen (T-Ag) and small t-antigen and the late region encodes VP1, VP2, VP3 and agnoprotein. The coding regions are transcribed in opposite directions from the NCCR, which is a bidirectional promoter containing the binding sites for the cellular and viral transcription factors that regulate JCV gene expression [11, 12].

Earlier work in our laboratory implicated the NF-κB signaling pathway and induction of expression of the Rad51 DNA repair protein as key regulators of the transcriptional status of JCV in the early stages of infection [13–18]. The JCV NCCR has a unique binding site for NF-κB and serves as a central nexus for regulation of JCV transcriptional activity. This site mediates modulation of JCV gene expression in response to a number of different stimuli including activation of the NF-κB signaling pathway by cytokines [14], other signal transducing transcription factors that cooperate or interfere with NF-κB stimulation such as NFAT4 [15] and C/EBPβ [13], as well as epigenetic regulatory events involving protein acetylation [16, 18]. Importantly, Rad51 expression is rapidly induced by JCV infection [19] and co-operates with NF-κB to stimulate JCV transcription and providing a positive feedback loop early in infection [17].

Rad51 is a key component of the cellular machinery for homologous recombination-directed double-strand break DNA repair that is the eukaryotic homologue of *Escherichia coli* RecA and highly conserved over many species [20, 21]. In an earlier study of JCV infection of primary human fetal astrocytes [19], we found a substantial induction of DNA damage and genomic instability reflected in changes in ploidy, increased micronuclei formation and an induction of the levels of phospho-histone2AX (γH2AX), a marker for double-strand breaks that recruits and targets multiple activities involved in DNA repair [22]. Concomitantly, JCV infection caused an induction in the expression levels of several enzymes involved in DNA repair, including a massive elevation in Rad51 expression [19]. In another other study, we found that Rad51 binds and activates the p65 subunit of NF-κB p65 and thus functions as a transcription factor [23, 24]. In the light of this observation, we next investigated a role for Rad51 on JCV transcription and replication and found that Rad51 activated transcription of the JCV early promoter [17]. This activation was co-operative with NF-κB p65 and could be abrogated either by mutation of the JCV NF-κB binding site or by co-expression of a dominant negative form of IκBα to sequester NF-κB indicating that Rad51 induction of JCV transcription operates through the NF-κB site [17]. Thus Rad51 and NF-κB are parts of a positive feedback mechanism that serves to enhance JCV gene expression during the early stage of viral infection.

In resting cells, NF-κB is sequestered in an inactive form in the cytoplasm by the inhibitory molecule, IκB, but can be released upon cellular stimulation allowing active NF-κB to enter the nucleus and stimulate the transcription of specific genes [25]. In the case of JCV, stimulation by extracellular proinflammatory cytokines such as TNF-α activates NF-κB by this pathway and strongly stimulates both early and late viral transcription, which may have a role in the reactivation of dormant virus [14]. As well as the canonical pathway of NF-κB activation, the DNA damage response (DDR) is also known to result in the activation of the NF-κB signaling pathway, a phenomenon known as nucleus to cytoplasm or “inside-out” NF-κB signaling [26–28], which is mediated by NF-κB essential modulator (NEMO), also known as IKK-γ, a subunit of the IκB kinase complex that mediates activation of NF-κB. Since JCV infection results in both the induction of Rad51 expression and DNA damage, it is possible that DDR-mediated nucleus to cytoplasm activation of the NF-κB pathway occurs and that crosstalk between these pathway allows them to act together to stimulate JCV transcription. In this study, we examine nucleus to cytoplasm NF-κB signaling in relation to JCV infection of glial cells.

## Methods

### Cell culture and plasmids

Culture of the human TC620 oligodendroglioma cell line and SVG-A, a cell line originally derived from human glial cells transformed by origin-defective SV40 that expresses SV40 T-Ag was as previously described [14, 29]. Expression plasmid pCMV-T-Ag expressing JCV T-antigen has been previously described [30]. Expression plasmids for human ATM and FLAG-tagged human NEMO are from Addgene (Cambridge, MA).

### Antibodies

Mouse monoclonal antibody to phospho-ATM (Ser1981, clone 10H11.E12) and rabbit monoclonal antibodies to total ATM (clone D2E2), Chk1 and phospho-Chk1(Ser 317) were from Cell Signaling Technology (Danvers, MA) and Chk2 and phospho-Chk2(Thr 68) from Santa Cruz Biotechnology, Inc. (Dallas, TX). Mouse anti-α-tubulin (clone B512) and anti-FLAG were from Sigma Aldrich (St. Louis, MO) and mouse anti-lamin A/C (clone 4C11) was from Cell Signaling Technology. Mouse monoclonal antibody to T-antigen (Ab-2 PAb416 DP02) was from Calbiochem (La Jolla, CA). Anti-VP1 antibody (AB597) was kindly provided by W. Atwood, Brown University, Rhode Island). Rabbit polyclonal anti-JCV agnoprotein antibody was previously described [31].

### Reagents

KU-55933(2-morpholin-4-yl-6-thianthren-1-yl-pyran-4-one), which is an ATP competitive inhibitor of ATM [32], CP466722 and doxorubicin were purchased from Sigma Aldrich (St. Louis, MO).

### Western blots

Cells were harvested as we have described [13, 33, 34] except for experiments to measure sumoylation where iodoacetemide (10 mM) was added to the lysis and wash buffers to preserve SUMOylation. Western blots were performed as described [33, 35, 36]. Briefly, 50 μg of protein was resolved by SDS-PAGE, transferred to nitrocellulose, and immunoblotted with primary antibody (1/1000 dilution) and secondary antibody (1/10000 dilution). Bound antibody was detected with the LI-COR system. Blots were incubated with IRDye® 680RD Goat Anti-Mouse Li-COR dyes and visualized with an Odyssey® CLx Imaging System (LI-COR, Inc., Lincoln, NE) using LI-COR Odyssey software. Band intensities were quantified using the Quantity One software (Bio-Rad, Hercules CA) and intensities normalized to loading control as previously described [34].

### Transient transfection

Transfection of expression plasmids was performed using Lipofectamine 2000 (Life Technologies Inc., Carlsbad CA) was performed as we have previously described [33, 34]. The total amount of transfected DNA was normalized with empty vector DNA.

### Immunoprecipitation(IP)/Western blot

TC620 cells were transfected with expression plasmids for FLAG-NEMO and JCV T-Ag, whole cell extracts prepared and IP/Western performed as we have described [33, 34]. ATM was immunoprecipitated followed by Western blot for FLAG or vice versa for the reciprocal IP/Western.

### MTT assay

To assess cell viability after treatment, the MTT (3-(4,5-dimethylthiazol-2-yl)-2,5-diphenyltetrazolium bromide) assay was performed with 5 mg/ml MTT (Amresco, Solon, OH) using an absorbance reader to determine optical density at a wavelength of 570 nm.

### JCV Infection

Infection experiments were performed with SVG-A cells. Cells were transfected with expression plasmids or treated with KU-55933 as specified in the respective Figure Legends and infected with Mad-1 JCV at an MOI of 1 as we have previously described [37, 38], harvested and analyzed after 7 days together with uninfected control cultures. Expression of the viral proteins VP1 and agnoprotein was measured in whole cell protein extracts by Western blot. In parallel, the growth medium of the cells was also collected to measure viral load by Q-PCR [38].

### Cell fractionation

SVGA cells were infected with Mad-1 JCV at an MOI of 1 and harvested at various time points after infection to isolate nuclear and cytoplasmic fractions using the NE-PER nuclear and cytoplasmic extraction kit according to the manufacturer’s instructions (Thermo Scientific Pierce, Rockford IL; Cat. #PI-78835) as we have previously described [17, 33, 35].

### Immunocytochemistry (ICC)

ICC was performed as we have recently described [17]. SVGA cells were infected with wild-type Mad-1 JCV and transfected twice with expression plasmid for FLAG-NEMO on days 2 and 5 postinfection. Cells were fixed in 4% paraformaldehyde, in PBS for 10 min, washed, permeabilized for 5 min with 0.1% Triton X-100, blocked with 5% normal goat serum for 30 min and incubated for 3 h at 37°C with mouse anti-FLAG or mouse anti-VP1 antibody at a 1:100 dilution in PBS. Cells were then washed, incubated for 2 h with secondary FITC-conjugated goat anti-mouse secondary antibody at a 1:200 dilution, washed, mounted with 4',6-diamidino-2-phenylindole (DAPI)-containing mounting medium (VECTASHIELD, Vector Laboratories Inc. Burlingame, CA), and viewed by fluorescence microscopy.

## Results

### JCV infection of glial cells results in phosphorylation of ATM

**Fig. 1:**
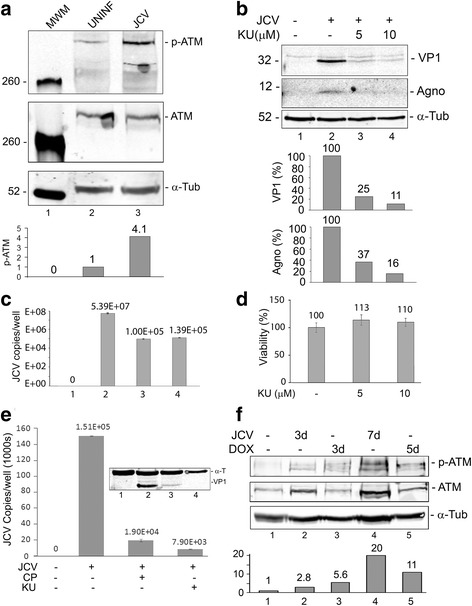
Role of ATM phosphorylation in JCV infection. **a** *Effect of JCV infection on ATM phosphorylation*. SVGA cells were uninfected or infected, as indicated, with the Mad-1 strain of JCV at moi = 1 for 5 days and harvested. Fifty micrograms of total cell extract were loaded onto a 6% polyacrylamide SDS gel, electrophoresed and analyzed by Western blot for phospho-ATM and total ATM. Alpha-tubulin (α-Tub) was the loading control. The intensity of the p-ATM band in each lane was quantified using the ImageQuant software (Molecular Dynamics, GE Healthcare Bio-Sciences, Pittsburgh PA) and these are shown in the lower part of the panel. **b** *Effect of ATM inhibition on JCV infection*. SVGA were infected with the Mad-1 of JCV and treated with or without KU-55933(2-morpholin-4-yl-6-thianthren-1-yl-pyran-4-one; 5 μM or 10 μM), which is ATP competitive inhibitor of ATM, as indicated. Cells were harvested and expression of VP1 and agnoprotein measured by Western blot. The loading control was α-Tubulin. The intensity of the VP1 and Agno bands in each lane was quantified using the ImageQuant software and these are shown in the lower part of the panel. **c** Culture supernatants from the cells in Panel **b** were assayed for virus using qPCR. Each viral load was measured in triplicate and presented as a histogram with the standard deviation shown as an error bar. **d** The viability of SVGA cells was assayed at different concentrations of KU-55933 by MTT assay as described in the Methods section. Each viability was measured in triplicate and presented as a histogram with the standard deviation shown as an error bar. **e** *Effect of different ATM inhibitors on JCV infection*. SVGA were infected with the Mad-1 of JCV and treated with or without KU-55933 or CP466722 as indicated. Cells and supernatants were harvested and viral copy number in the culture medium assayed by QPCR. Expression of VP1 was measured by Western blot with α-Tubulin as the loading control (shown inset). **f** *Effect of JCV infection or doxorubicin treatment on ATM phosphorylation*. SVGA cells were uninfected or infected, as indicated, with the Mad-1 strain of JCV at moi = 1 or treated with 2.5 μM doxorubicin for the times indicated and harvested. Western blots were performed for phospho-ATM and total ATM. Alpha-tubulin was the loading control. The intensity of the p-ATM band in each lane was quantified using the ImageQuant software and these are shown in the lower part of the panel.

The first step in nucleus to cytoplasm NF-κB signaling is the activation and autophosphorylation of ATM, a serine/threonine-specific protein kinase that is recruited and activated by double-strand DNA breaks. It phosphorylates several key signaling proteins that initiate cellular responses to DNA damage including p53, CHK2, BRCA1, NBS1, H2AX and NEMO [39]. First, we examined the phosphorylation of ATM Ser1981, the serine residue upon which ATM is autophosphorylated in response to DNA damage [40]. As shown in Fig. 1a, Western blot with a phosphospecific antibody to ATM showed that phospho-ATM is increased 5 days after JCV infection of SVGA cells. Total ATM and the loading control α-tubulin were unchanged.

### ATM inhibition restrains JCV infection of glial cells

To further investigate the role of ATM in JCV infection, SVGA were infected with JCV and treated with or without KU-55933, which is an ATP competitive inhibitor of ATM. Culture supernatants were assayed for virus by qPCR and cells were harvested at different time points and samples were used for Western blots for viral proteins. KU-55933 caused a reduction in viral protein expression compared to control (Fig. 1b) and likewise a reduction in the virus load in the culture supernatants of infected cells (Fig. 1c). At the concentrations used, KU-55933 had no toxic effects on cells as measured by MTT assay (Fig. 1d). To verify the effect of ATM inhibition, we also employed CP466722, a second inhibitor of ATM structurally unrelated to KU-55933. CP466722 suppressed viral copy number down from 1.5 × 10^5^ to 1.9 × 10^4^ compared to 0.8 × 10^4^ for KU-55933 (Fig. 1e).

**Fig. 2:**
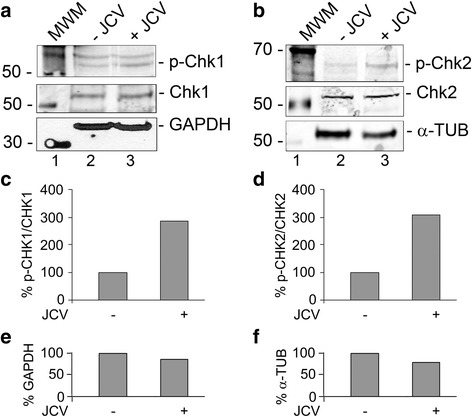
Phosphorylation of CHK1 and CHK2 in JCV infection. SVGA cells were infected with Mad-1 JCV at an moi of 1 and cultured for 5 days. Total cell lysates were harvested and analyzed by Western blots for phospho-Chk1(Ser 317), Panel **a** and phospho-Chk2(Thr 68), Panel **b**. The Western membranes were reprobed for total Chk1 (**a**) and Chk2 (**b**) and then for a loading control, which was either glyceraldehyde-3-phosphate dehydrogenase (GAPDH, **a**) or α-tubulin (α-TUB, **b**). The intensity of each band was quantified using the IMAGEQUANT software (Molecular Dynamics, GE Healthcare Bio-Sciences, Pittsburgh PA) and the ratio of phospho-Chk1 to Chk1 (**c**) and phospho-Chk2 to Chk2 (**d**) calculated and shown as histograms expressed as a percentage of the signal in uninfected cells. Similarly, the loading controls were quantified (**e** and **f**)

### Comparison of JCV infection of glial cells to treatment with a chemical activator of the DDR on the phosphorylation of ATM

To compare to the induction phospho-ATM in response to JCV infection to treatment with doxorubicin, a known chemical activator of the DDR, SVGA were infected with JCV for 3d and 7d or treated with doxorubicin for 3d and 5d (7d treatment was toxic to the cells). Both JCV infection and treatment with doxorubicin caused induction phospho-ATM of a comparable order of magnitude (Fig. 1f).

### JCV infection causes the phosphorylation of proteins in the ATM kinase signal transduction pathway

The activation of the serine/threonine-specific protein kinase, ATM, is the upstream event in a signal transduction pathway, which is initiated in response to DNA damage. In order to further explore the activation of the DNA damage response, we investigated the phosphorylation/activation status of the intermediate protein kinases Chk1 (Fig. 2a) and Chk2 (Fig. 2b) in this pathway using phospho-specific antibodies. For each, the amount of total Chk1 and Chk2 protein was also measured and phosphorylation was normalized to this (Fig. 2c & d). Additionally, the levels of loading controls were measured for each Western by quantifying expression of glyceraldehede-3-phosphate dehydrogenase (Fig. 2e) or α-tubulin (Fig. 2f). The levels of Chk1 and Chk2 phosphorylation were increased by 2.9-fold and 3-fold respectively when cells were infected by JCV. Thus both Chk1 and Chk2 are activated by JCV infection.

### JCV infection causes the subcellular redistribution of NEMO to the nucleus as determined by subcellular fractionation

**Fig. 3:**
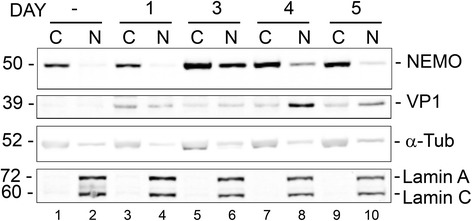
Role of NEMO cytoplasm to nucleus translocation in JCV infection: Analysis of the effect of JCV infection on the subcellular distribution of NEMO by cell fractionation. SVGA cells were transfected with expression plasmid for FLAG-NEMO and infected with wild-type Mad-1 JCV 24 h later. Cells were harvested at 1, 3, 4 and 5 d after infection and fractionated into cytoplasmic and nuclear fractions. Western blot for α-FLAG was used to monitor the subcellular distribution of NEMO. Western blots for α-tubulin and lamin A/C were used to monitor cell fraction purity and for VP1 to monitor infection

Downstream of ATM, there are a series of post-translational modifications of the NF-κB essential modulator (NEMO), which occur after NEMO is translocated to the nucleus [41]. To investigate NEMO translocation, SVGA cells were transfected with expression plasmid for FLAG-NEMO, infected with JCV and harvested at various time points after infection to isolate nuclear and cytoplasmic fractions. Western blot for α-FLAG showed that NEMO underwent translocation from the cytoplasm to the nucleus, which was maximal at 3 days following infection (Fig. 3). The integrity of the cytoplasmic and nuclear fractions was verified by Western blot for α-tubulin and lamin A/C respectively. Infection was monitored by Western blot for VP1.

### JCV infection causes the subcellular redistribution of NEMO to the nucleus as determined by immunocytochemistry

**Fig. 4:**
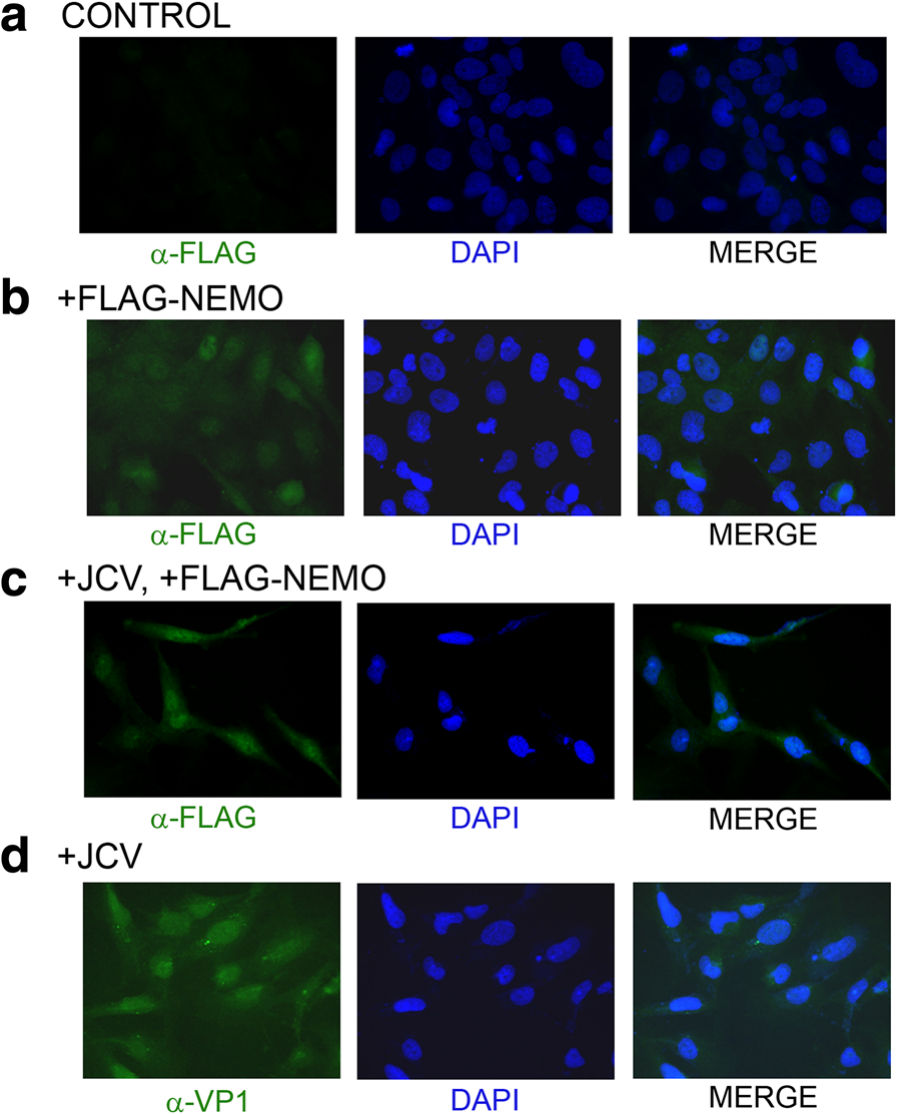
Role of NEMO cytoplasm to nucleus translocation in JCV infection: Analysis of the effect of JCV infection on the subcellular distribution of NEMO by immunocytochemistry. SVGA cells were uninfected and untransfected (**a**) or uninfected and transfected with expression plasmid for FLAG-tagged NEMO (**b**). SVGA cells were also infected with wild-type Mad-1 JCV and transfected twice with expression plasmid for FLAG-NEMO on days 2 and 5 postinfection (**c**). Control cells were infected with JCV but not transfected (**d**). Immunocytochemistry for FLAG and VP1 was performed on day 7 postinfection as described in the Methods section

In another experiment, NEMO translocation was investigated by immunocytochemistry. SVGA cells were infected with Mad-1 JCV and transfected twice with FLAG-NEMO (days 2 and 5) and analyzed by immunocytochemistry for FLAG and VP1 on day 7. As shown in Fig. 4, the distribution of NEMO was revealed by an α-FLAG antibody (FITC, green) and nuclei by DAPI (blue). It was again found that NEMO translocates from the cytoplasm to the nucleus upon JCV infection (compare Fig. 4b and c).

### JCV large T-Ag causes the sumoylation of NEMO

**Fig. 5:**
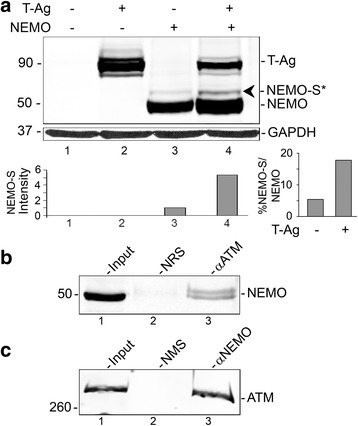
Role of NEMO sumoylation in JCV infection and NEMO association with ATM. **a** Effect of JCV T-antigen on Sumoylation of NEMO. TC620 cells were transfected with expression plasmids for FLAG-tagged NEMO and/or T-Ag. Western blots were performed with antibody to T-Ag and anti-FLAG antibody to detect NEMO and sumoylated NEMO (arrowhead) as indicated. The quantified intensity of the sumoylated NEMO band is indicated in the histogram directly below the Western. The intensity of the sumoylated band as a percentage of total NEMO with and without T-Ag is presented in the lower right-hand part of the panel. **b** *Physical association between ATM and NEMO*. TC620 cells were transfected with expression plasmids for ATM and FLAG-NEMO. Whole cell extract (Input, lane 1) was immunoprecipitated with rabbit anti-ATM (lane 3). Nonimmune rabbit serum (NRS) was the negative control (lane 2). Western blot was with α-FLAG antibody. **c** In a reciprocal immunoprecipitation, whole cell extract from Panel **b** (Input, lane 1) was immunoprecipitated with mouse anti-FLAG (lane 3). Nonimmune mouse serum (NMS) was the negative control (lane 2). Western blot was with α-ATM antibody

After translocation to the nucleus, NEMO undergoes post-translational modification by the covalent attachment of SUMO and ubiquitin [41]. In the next experiment, we transfected cells with expression plasmids for FLAG-tagged NEMO with or without co-transfection of an expression plasmid for JCV T-Ag. Western blots were performed with antibody to the FLAG epitope to detect unmodified NEMO and NEMO, which is sumolyated and/or ubiquitinated and thus migrates at a higher molecular weight (Fig. 5a). Both unmodified and modified forms of NEMO were detected in cells transfected with FLAG-NEMO and modified NEMO was increased about 5.5-fold in the presence of JCV T-Ag indicating that T-Ag is involved in NEMO sumoylation/ubiquitination.

### ATM and NEMO physical associate with each other

In the next step of nucleus to cytoplasm NF-κB activation, NEMO associates with activated ATM and ATM phosphorylates NEMO to promote its ubiquitin-dependent nuclear export [42]. To investigate physical association between ATM and NEMO, we transfected cells with expression plasmids for ATM and FLAG-NEMO and used whole cell extract for immunoprecipitation with an antibody to ATM. Western blot with α-FLAG antibody to detect NEMO, showed that NEMO had co-immunoprecipitated with ATM but not in the nonimmune rabbit serum (NRS) negative control (Fig. 5b). Reciprocally when whole cell extract was immunoprecipitated with mouse anti-FLAG to immunoprecipitate NEMO, ATM was co-immunoprecipitated but in the nonimmune mouse serum (NMS) negative control (Fig. 5c). We conclude that ATM and NEMO physically associate with each other.

## Discussion

Here we report that infection of glial cells by JCV activates the nucleus to cytoplasm or “inside out” pathway of NF-κB signaling. The first step in this pathway is activation of the ATM by DNA damage serine/threonine-specific protein kinase, which can be measured by phosphospecific Western blot for the autophosphorylated form of the protein (Ser1981) that is the activated form [39]. This finding was not surprising since we have already reported that phosphorylation of H2AX, a downstream target of ATM in the DDR, occurs after JCV infection [19]. We also investigated the phosphorylation of two other signaling intermediates downstream of ATM, the checkpoint kinases Chk1 and Chk2 and found that they are both activated by phosphorylation on serine 317 (Chk1) and threonine 68 (Chk2).

A key step downstream of ATM in nucleus to cytoplasm NF-κB activation is the translocation of NEMO to the nucleus and its sumoylation. We now report that both of these events occur after JCV infection. Interestingly, it is known that NEMO translocation and sumoylation is independent of ATM activation [41]. Indeed, other stress conditions, including oxidative stress, ethanol exposure, heat shock and electric shock, also induce NEMO SUMOylation [43]. Thus DNA damage per se is not necessary for sumoylation of NEMO to occur. The nature of the stress incurred by JCV infection that induces NEMO sumoylation is not known but it is possible that the oncogenic stress arising from the disruption of multiple cellular signaling pathways by T-Ag expression may be involved [44] in addition to the effect of DNA damage.

**Fig. 6:**
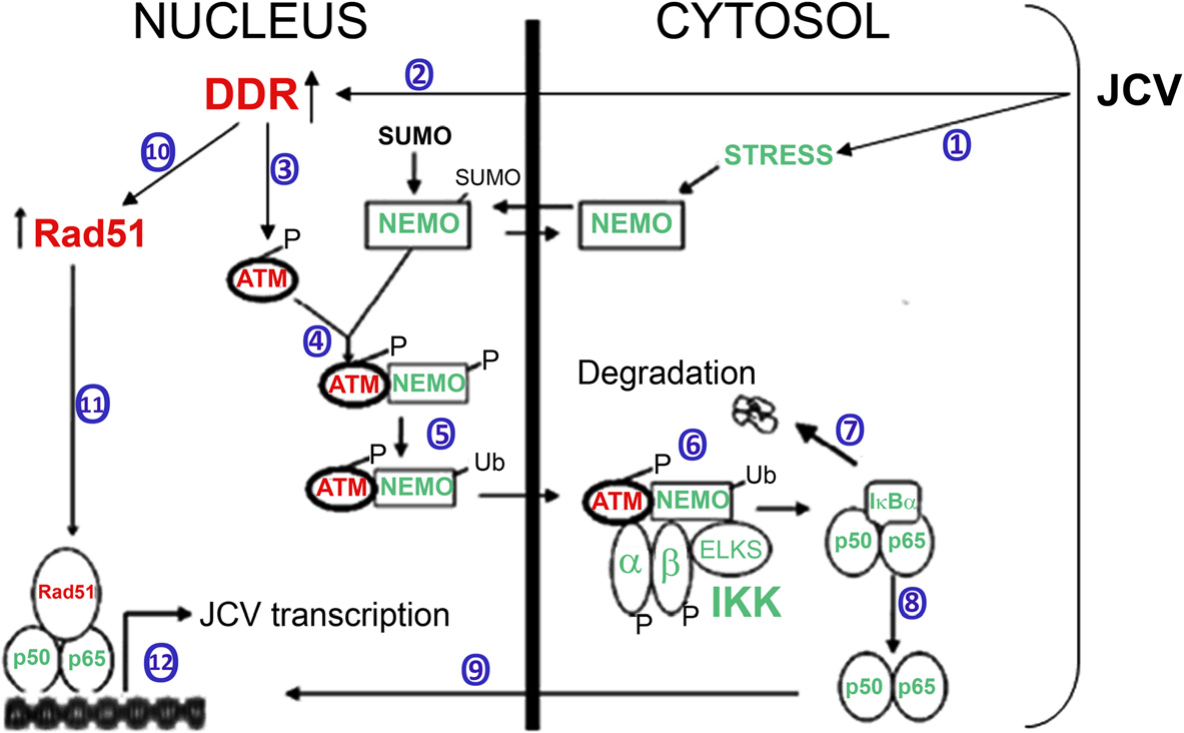
Schematic representation of the nucleus to cytoplasm NF-κB activation pathway in JCV infection. Infection of glial cells by JCV results in stress leading to NEMO sumoylation in the nucleus ① and activation of the DDR ②, which phosphorylates and activates ATM ③. Activated ATM binds NEMO and leads to phosphorylation of NEMO on Ser85 ④, which is involved in NEMO ubiquitination and export from the nucleus ⑤ to the cytoplasm. ATM and NEMO activate IKK ⑥, which phosphorylates IκB leading to IκB degradation ⑦ and release of NF-κB ⑧ to migrate to the nucleus ⑨. Meanwhile, Rad51 is induced by the DDR ⑩ and associates with nuclear NF-κB ⑪ to activate JCV transcription ⑫

While the NEMO sumoylation step is ATM-independent, it causes NEMO to be retained in the nucleus and allows subsequent ATM-dependent phosphorylation and ubiquitylation of NEMO to ultimately activate cytoplasmic IKK [41]. A schematic model of the DDR and NF-κB pathways and their cross-interaction in regulating JCV is shown in Fig. 6.

From earlier studies, it is known that active NF-κB translocation to the nucleus [45, 46] stimulates transcription of NF-κB-dependent genes including both the early and late genes of JCV as described in our earlier studies [13–18, 35, 36]. JCV infection-induced DDR signaling is associated with a large elevation in the expression level of Rad51 protein [19], which then physically associates with NF-κB and augment its function [23] and stimulate transcription of JCV early gene expression [17]. Activation of JCV transcription by Rad51 is abrogated by mutation of the NF-κB binding site or addition of siRNA to NF-κB p65 [17]. This indicates that Rad51 induction resulting from JCV infection with JCV acts through NF-κB to stimulate JCV early transcription. Since we now show NF-κB activation is itself induced by JCV infection, this suggests the operation of a novel two-part positive feedback mechanism that enhances viral gene expression early in infection.

The induction of the DDR by infection may be a general feature of polyomaviruses [47] as indeed is also the case for many other types of both DNA and RNA viruses [48–51]. The closely related BKV has also been reported to induce the DDR: infection of the natural host cells of the virus, renal proximal tubule epithelial (RPTE) cells, activated both the ATM and the ATR (ATM and Rad3-related) DDR [52]. Knockdown of ATM or ATR by RNA interference reduced BKV replication and virion production while double knockdown had an additive effect indicating that DDR induction is critical for productive BKV infection [52]. Further analysis revealed that neither input virus nor expression alone was sufficient to trigger ATM or ATR activation but rather ATM- and ATR-mediated DDR is associated with viral DNA replication [53]. For the rhesus macaque monkey polyomavirus SV40, infection of permissive CV1 or BSC40 monkey cells causes activation of ATM signaling [54, 55], H2AX phosphorylation and Mre11-Rad50-Nbs1 assembly with T-antigen together with other DDR-signaling proteins in viral replication foci. Inhibition of ATM kinase showed that this is a prerequisite for optimal viral replication [55]. Hein et al. [56] found that expression of SV40 large T-antigen alone, without a viral replication origin, induced DDR markers including γ-H2AX, p53 nuclear foci and other signaling components downstream of ATM and ATR kinases such as CHK1 and CHK2. Large T-antigen also induced apparent DNA damage as measured by COMET assay, the Fanconi anemia pathway and Rad51 foci [57]. It was further demonstrated using Rad51 siRNA that Rad51 is required for efficient viral replication [57] similar to the role of Rad51 that we have reported in JCV replication [17]. Activation of the ATM pathway has also been reported for mouse polyomavirus [58] and human Merkel cell carcinoma virus [59]. In addition to polyomaviruses, DDR activation as a general feature of other viruses including herpesviruses [60] and papillomaviruses [61]. For example, Kaposi sarcoma-associated herpes virus (KSHV) expresses genes which selectively modulate the DDR through the activation of the ATM pathway [60] and human papillomavirus (HPV) activates both the ATM and ATR DDR pathways [61].

In the case of JCV, besides our study described here, there is one other report of the activation of ATM by viral infection [62]. In this report, infection by JCV or transient transfection with a T-Ag expression plasmid resulted in activation of ATM and ATR causing cells to accumulate in the G_2_ phase of the cell cycle. Inhibition of ATM and ATR by caffeine suppressed JCV replication and virus production [62]. These studies showed the role of ATM and ATR signaling in the JCV life cycle. However, our study described here is the first to report the involvement of the nucleus to cytoplasm pathway of NF-κB activation downstream of ATM by JCV or by any other polyomavirus.

## Conclusions

In summary, our results reported here describe the novel finding that DNA damage following JCV infection by JCV activates ATM and initiates nucleus to cytoplasm NF-κB signaling. In this way, JCV is able to enhance expression of its own genes and promote the viral life cycle, which may be important in the pathogenesis of PML since it provides potential positive feedback during viral reactivation.
